# Immunotherapy in the context of sepsis-induced immunological dysregulation

**DOI:** 10.3389/fimmu.2024.1391395

**Published:** 2024-05-21

**Authors:** Yiqi Wu, Lu Wang, Yun Li, Yuan Cao, Min Wang, Zihui Deng, Hongjun Kang

**Affiliations:** ^1^ Department of Critical Care Medicine, The First Medical Center, Chinese People’s Liberation Army (PLA) General Hospital, Beijing, China; ^2^ Graduate School of The People’s Liberation Army (PLA) General Hospital, Beijing, China; ^3^ Department of Emergency Medicine, The Second Hospital of Hebei Medical University, Shijiazhuang, China; ^4^ Department of Basic Medicine, Graduate School, Chinese PLA General Hospital, Beijing, China; ^5^ National Key Laboratory of Kidney Diseases, National Clinical Research Center for Kidney Diseases, Beijing Key Laboratory of Kidney Disease Research, Beijing, China

**Keywords:** sepsis, immunological dysregulation, immunotherapy, immunostimulatory therapy, immunosuppressive therapy

## Abstract

Sepsis is a clinical syndrome caused by uncontrollable immune dysregulation triggered by pathogen infection, characterized by high incidence, mortality rates, and disease burden. Current treatments primarily focus on symptomatic relief, lacking specific therapeutic interventions. The core mechanism of sepsis is believed to be an imbalance in the host’s immune response, characterized by early excessive inflammation followed by late immune suppression, triggered by pathogen invasion. This suggests that we can develop immunotherapeutic treatment strategies by targeting and modulating the components and immunological functions of the host’s innate and adaptive immune systems. Therefore, this paper reviews the mechanisms of immune dysregulation in sepsis and, based on this foundation, discusses the current state of immunotherapy applications in sepsis animal models and clinical trials.

## 1 Introduction

Sepsis is defined as a life-threatening organ dysfunction caused by a dysregulated host response to infection, which can progress to septic shock and/or multiple organ dysfunction or failure in severe cases (1). Recently, sepsis has exhibited characteristics of “three highs and one low,” namely high incidence, high mortality, high disease burden, and low recovery rates. Rudd et al. reported approximately 48.9 million cases of sepsis globally and 11 million sepsis-related deaths in 2017, accounting for 19.7% of all global deaths (2). Li et al. identified 9,455,279 registered hospital cases of sepsis in China from 2017 to 2019, with 806,728 related deaths, particularly among high-risk groups such as children under the age of 9 (incidence rate: 20.4%) and the elderly aged 65 and older (incidence rate: 57.5%) (3). Furthermore, a recent study estimated the annual direct and indirect economic costs associated with sepsis in the Netherlands to be between 3.8 and 6.5 billion euros (4). Moreover, survivors of sepsis from different demographics may experience various adverse outcomes, such as long-term neurodevelopmental abnormalities and physical dysfunctions in neonates, as well as cognitive impairments and psychological issues such as depression and anxiety (5–8). These phenomena are not only related to the rapid progression and unpredictable nature of the complex clinical picture but also due to the lack of specific therapeutic measures in current clinical practice. Undoubtedly, the early implementation of a sepsis bundle strategy, including fluid resuscitation, antibiotic therapy, and lung-protective ventilation, is necessary and crucial. These protective measures have a positive impact on patients with sepsis and have significantly reduced mortality rates to some extent (9–11). However, studies have also found that inappropriate fluid management (12) and uncontrolled use of broad-spectrum empirical antibiotics (13–15) may hinder the timely control of the patient’s condition. Additionally, the management of sepsis patients should also focus on modulating the host response, including but not limited to the use of corticosteroids and vasopressors (16).

As early as 1893, William Coley inadvertently discovered that postoperative infections with pyogenic *Streptococcus* could induce tumor regression in sarcoma patients (17), thereby unveiling the prelude to immunotherapy. With the rapid advancement of biomedical technologies, various autologous immunotherapies (such as dendritic cells (DCs), interleukin (IL)-2) (18–20), genetically engineered therapies (such as CAR-T therapy) (21, 22), and recombinant antibodies (such as bispecific antibodies, trispecific antibodies) (23–25) have been developed and introduced into clinical trial. Although immunotherapy was initially and most extensively applied in the field of oncology, as research has revealed that virtually all diseases have some form of direct or indirect relation with the immune system, the scope of immunotherapy has extended beyond cancer treatment. It now encompasses therapies aimed at inducing, enhancing, or suppressing the patient’s own immune response to treat a wide array of diseases related to immune molecules, immune cells, and the immune system itself, including infectious diseases (such as sepsis) (26), autoimmune diseases (such as systemic lupus erythematosus) (27, 28), and diseases related to immunosenescence and inflammaging (such as atherosclerosis, Alzheimer’s disease, and diabetes) (29–32). It is commonly accepted that the core pathophysiological mechanism of sepsis involves a dysregulated immune response characterized by acute-phase hyperinflammation followed by late-phase immune suppression, triggered by pathogens. This suggests the feasibility of immunotherapy (33). Compared to bundle therapies, immunotherapy can modulate disease through mechanisms such as cytokine level adjustment, targeting immune checkpoints/blocking programmed cell death, and supplementing immunoglobulins. This adjusts the components and functions of the patient’s innate and adaptive immune systems, thereby facilitating the rapid restoration of immune homeostasis. Additionally, with the continuous development of novel biomarkers (34, 35), medical professionals may soon be able to track and record changes in a patient’s immune function in real-time by analyzing immune components, metabolic products, differentially expressed proteins or genes in bodily fluids or tissues. This development could provide convenient management pathways and monitoring windows for immunotherapy. Of course, potential side effects and immune-related adverse events (IR-AEs) during treatment should not be overlooked. This underlines the importance of developing convenient and reliable markers to dynamically monitor patients’ immune statuses during immunotherapy.

Although recent research into the use of immunotherapy for treating sepsis has made preliminary progress, issues such as the therapy’s stability, long-term efficacy, and potential side effects remain to be validated. This article reviews the mechanisms of immunological dysregulation in sepsis hosts and summarizes the application of immunotherapy in both sepsis animal models and clinical patients based on this understanding. Additionally, we present perspectives on the prospects and challenges of applying immunotherapy in the treatment of sepsis.

## 2 Sepsis-induced immunological dysregulation

Initially, sepsis was believed to be primarily driven by an excessive systemic inflammatory response induced by exogenous and/or endogenous infections. However, increasing evidence suggests that during the progression of sepsis, the phenotype of the host’s immune cells can shift from pro-inflammatory to anti-inflammatory, with a rise in anti-inflammatory cytokine levels and a marked reduction in pro-inflammatory cytokines. In the later stages of the disease, there is even sustained apoptosis of immune cells, indicating that “sepsis is not merely an inflammatory response.” Furthermore, some clinical trial results have reported that immunosuppressive therapies have few therapeutic effect on sepsis patients, underscoring that sepsis is not simply a process of inflammatory response. Today, a multitude of preclinical and clinical studies have confirmed that sepsis involves a complex syndrome with multiple intrinsic mechanisms, including systemic inflammatory response syndrome (SIRS), compensatory anti-inflammatory response syndrome (CARS), immunoparalysis, inflammatory cytokine gene remodeling, and endotoxin tolerance (ET).

**Figure 1:**
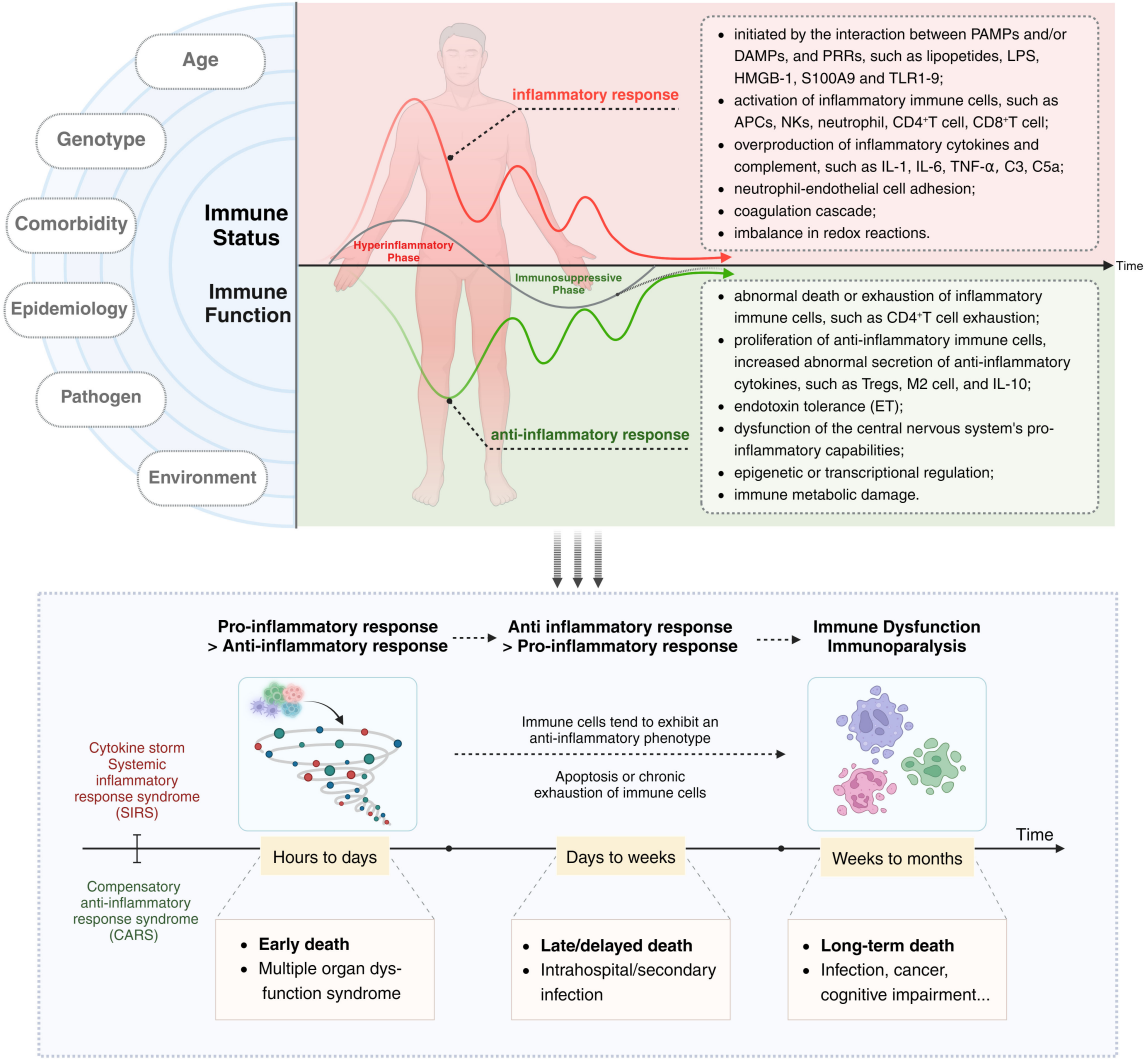
The immune dysregulation mechanism and related characteristics of sepsis: Current research supports the notion that both SIRS and CARS occur simultaneously at the onset of sepsis. However, an imbalance in the intensity and duration of responses between these two phases leads to clinical manifestations characterized initially by an excessive inflammatory response and later by immune suppression or immune paralysis. Acute excessive inflammatory response is associated with early death, while later immune paralysis/tolerance is an important cause of late or long-term death. The left side of the diagram delineates factors influencing the host’s immune status and function, such as age, genotype, comorbidities, epidemiology, pathogen type, and environmental conditions. The central and right portions of the diagram depict the immune status of a host with sepsis, with the upper half focusing on excessive inflammatory responses and their associated characteristics, and the lower half detailing secondary immune suppression and its related features. The black arrow (“*Time*”) indicates that the immune response and immune status of sepsis patients undergo dynamic and imbalanced changes over time/disease progression.

Current research supports the notion that both SIRS and CARS occur simultaneously at the onset of sepsis (36, 37). However, an imbalance in the intensity and duration of responses between these two phases leads to clinical manifestations characterized initially by an excessive inflammatory response and later by immune suppression or immune paralysis. During this period, factors such as the host’s age, ethnicity, genetic background, comorbidities, and the type of pathogen can influence the immune status and clinical features of sepsis patients. In terms of overall effects, the acute phase of the disease course in sepsis hosts is marked by excessive activation of immune cells, cytokine storm (CS), and SIRS, while the later stages are characterized by increased immune cell apoptosis or chronic exhaustion and functional impairment, as well as ET (**Figure 1**).

### 2.1 The acute phase is dominated by an excessive inflammatory response

During the acute phase of sepsis, both in animal models and in patients, a significant elevation is observed in white blood cell counts and levels of inflammatory cytokines, such as tumor necrosis factor (TNF)-α, interferon (IFN)-γ, and IL-6 (38–41). When the host is exposed to exogenous and/or endogenous pathogens’ pathogen associated molecular patterns (PAMPs), such as lipopolysaccharide (LPS) (42, 43) and mannose-binding lectin (MBL) (44), or to damage-associated molecular patterns (DAMPs) released from its own damaged tissue cells, such as histones (45), high-mobility group box-1 (HMGB-1) (46, 47), and heat shock proteins (HSPs) (48), these molecules are recognized and interact with pattern recognition receptors (PRRs) on the surface of antigen-presenting cells. This interaction marks the initiation of the acute inflammatory response in sepsis. Current research has categorized PRRs into five subfamilies: the Toll-like receptors (TLRs) (49, 50), the nucleotide oligomerization domain (NOD)-like receptors (NLRs) (51, 52), the retinoic acid-inducible gene-I (RIG-I)-like receptors (RLRs) (53), the C-type lectin receptors (CLRs) (54, 55), and the Absent in melanoma-2-like receptors (ALRs) (56). The effector domains of PRRs mediate the activation of downstream inflammatory signaling pathways by recognizing specific ligands. This activation leads to the release of pro-inflammatory cytokines, recruitment of innate immune cells, and induction of inflammatory responses. Specifically, to address intracellular infections caused by pathogens, certain cytoplasmic PRRs (typically from the NLRs or ALRs families) often serve as receptor proteins that participate in the assembly of inflammasomes. Inflammasomes recruit pro-caspase-1 and activate it to caspase-1, which in turn cleaves pro-interleukin-1β, pro-interleukin-18, and gasdermin (GDSMD) into their mature forms, triggering an inflammatory response and cell pyroptosis. Subsequently, these cytokines induce further proliferation and activation of immune cells, skewing them toward an inflammatory phenotype and elevating levels of various inflammatory mediators such as interleukins IL-1, IL-6, IL-17, TNF-α, IFN-γ, and chemotactic factors such as prostaglandins, histamine. For instance, neutrophils combat pathogens through direct actions such as phagocytosis, degranulation, and the release of neutrophil extracellular traps (NETs), as well as indirectly through the release of reactive oxygen species (ROS), reactive nitrogen species (RNS), and proteolytic enzymes during cell proliferation and migration (57–60). Macrophages polarize toward an M1 inflammatory phenotype and release large amounts of IL-1β, TNF-α, and IL-6 (61–63), while DCs mediate the activation of CD4^+^T (64). This syndrome is also accompanied by an imbalance of redox reactions (65, 66), neutrophil-endothelial cell adhesion (67), activation of the complement system (68, 69), and the coagulation cascade (70, 71). Additionally, intense complement activation (especially C3 and C5a) enhances vascular permeability, and increases the adhesiveness between leukocytes and vascular endothelial cells, further promoting the inflammatory response and damaging self-tissue organs (72, 73). A recent single-cell transcriptomic analysis revealed the inflammatory profile of peripheral blood mononuclear cells (PBMCs) in COVID-19 and sepsis patients, identifying ten highly inflammatory cell subtypes and their characteristics (74). The adaptive immune response typically lags behind the innate immune response, with antigen-presenting cells activating T lymphocytes through the dual signaling system of the major histocompatibility complex (MHC)/antigen peptide-T cell receptor (TCR) and CD80-CD28 (75, 76). Under the influence of various cytokines released by innate immune cells, tissue cells, and activated lymphocytes (77), T lymphocytes differentiate into multiple subgroups, including cytotoxic T cells (CTLs), helper T cells (Th), and regulatory T cells (Tregs), playing a crucial role in the acute phase inflammatory response in sepsis hosts. Additionally, B lymphocytes counteract pathogen invasion by secreting antigen-specific antibodies and inflammatory cytokines (78).

Unfortunately, during the acute phase of sepsis, the excessive activation of immune cells and pro-inflammatory cytokines is not adequately restrained by anti-inflammatory responses. Consequently, this uncontrolled and excessive inflammatory response not only fails to efficiently eradicate pathogens within the host but also leads to severe cellular death, tissue damage, and organ dysfunction, potentially resulting in the early death of the host (79).

### 2.2 The later stages are characterized by secondary immune suppression

Under normal circumstances, as infections are cleared and host compensatory anti-inflammatory responses are modulated, a patient’s heightened inflammatory response gradually subsides and returns to physiological levels, ultimately restoring the immune homeostasis of the internal environment. However, in the later stages of sepsis, the host often experiences a dysregulated anti-inflammatory response, which is not conducive to moderating inflammation but instead manifests as immune tolerance or paralysis, showing a low response to pathogens (80). These factors significantly increase the risk of secondary infections and adverse prognoses, including late or posthumous deaths (81, 82).

Lymphocyte apoptosis or reduction in their numbers is a significant factor contributing to immune paralysis. Preclinical studies using sepsis animal models indicate widespread apoptosis of parenchymal and immune cells across multiple organs, including the thymus, spleen, lungs, intestines, and skeletal muscle (83–86). More importantly, in mice undergoing cecal ligation and puncture (CLP), it has been observed that sepsis-induced impairment in T cells’ ability to combat pathogen infections can persist for several months (87). In clinical research, uncontrolled circulating immune cell apoptosis is considered a primary cause of impaired immune function (88–90). Additionally, transcriptomic analysis revealed that circulating lymphocytes in the later stages of the disease exhibit low inflammatory activity and immune suppression (91). There is a notable reduction in the numbers of CD4^+^ and CD8^+^T cells, with an increased proportion of Tregs (90, 92). This impaired proliferative capacity and sustained apoptosis of T lymphocytes may be associated with the upregulation of negative signaling pathways, such as the programmed death receptor-1/programmed death ligand-1 (PD-1/PD-L1) axis (93–96). The role of B lymphocytes in immune suppression remains unclear, yet studies have identified selective depletion or increased apoptosis of memory B cells in sepsis patients (97). These cells exhibit reduced MHC II expression and tend toward a CD21^low^CD95^high^ exhausted-like phenotype (98), with a significant reduction in antigen-specific antibody release. During the process of immune suppression in the host, innate immune cells are both victims and perpetrators (99–102), including neutrophils (103–105), DCs (106–108), and monocytes/macrophages (109–112). These cells commonly experience abnormal differentiation, functional impairments, and extensive tissue infiltration. Research has shown that an increased proportion of immature neutrophils (CD10^low^CD16^low^ cells) in the whole blood of sepsis patients is associated with an increased risk of early death within 48 hours after sepsis onset (113). This may be linked to the upregulation of myeloid-derived suppressor cells (MDSCs) subgroups (113) and the integrin Mac-1 (αMβ2) (114), which mediates suppression of T cell proliferation. Recent studies have reported a higher abundance of immature neutrophil subgroups expressing genes related to *IL1R2*, *PADI4*, and *MPO* in sepsis patients, and *in vitro* experiments suggest that these immature neutrophils can inhibit the proliferation and activation of CD4^+^T cells (115). Significant changes also occur in DCs, characterized by the acute phase’s systemic high-inflammatory microenvironment excessively activating the immature DCs stored in parenchymal tissues and lymphoid organs until they are exhausted. However, newly generated DCs are functionally immature, which includes acquiring the immunogenic phenotype of pathogens, capturing, processing, and/or presenting antigens, as well as the capacity to stimulate T cell activation (116). Recent studies have found that low expression of monocyte human leukocyte antigen DR (_M_HLA-DR) of in sepsis patients reduces activation of T lymphocytes (117). Increasing evidence suggests that low expression of _M_HLA-DR can serve as a biomarker for predicting immune paralysis or poor prognosis in sepsis (118–121). Concurrently with extensive apoptosis of immune cells, a class of immature myeloid cells collectively known as MDSCs—which include progenitors or precursors of neutrophils, DCs, and monocytes—proliferate abundantly and are released into the bloodstream (122, 123). These MDSCs exhibit significant immunosuppressive properties, including inhibiting the proliferation and activation of effector lymphocytes while activating Tregs, reducing the production of inflammatory cells and promoting the release of anti-inflammatory cytokines, as well as upregulating the expression of immune checkpoint molecules.

Endotoxin tolerance constitutes a critical aspect of immunosuppression (124), often resulting from the innate and adaptive immune cells of the host being persistently exposed to low levels of endotoxins or LPS, entering a transient “desensitized state.” This leads to an unresponsive state of the host immune system to sudden, high-dose endotoxin or LPS exposure. Numerous preclinical studies have shown that mouse monocytes continuously exposed to LPS *in vitro* can undergo ET, characterized by the downregulation of inflammatory cytokines (including TNF-α, IL-6, and IL-8) and the upregulation of anti-inflammatory cytokine (IL-10) expression (125). Macrophages from mice pretreated with LPS also exhibit a diminished response to subsequent LPS stimulation *in vitro*, with downregulated mRNA expression of genes encoding recombinant *granulocyte-macrophage colony-stimulating factor* (*GM-CSF*), *IFN-γ-inducible protein-10*, *JE/monocyte chemoattractant protein-1*, and *macrophage-inflammatory protein-1β/2 (* 126). This reduced responsiveness and immune tolerance of monocytes/macrophages to LPS stimulation may be associated with the remodeling of NF-κB function, chromatin modifications, and reprogramming of inflammatory genes (127). Besides, tolerance-inducing DCs in mice, which express lower levels of MHC-II and CD86, can induce the proliferation and recruitment of CD4^+^Foxp3^+^ Tregs through the secretion of TGF-β (128). In clinical research, healthy volunteers continuously stimulated with LPS exhibited a downregulation of pro-inflammatory cytokine levels *in vivo (* 129). *In vitro* experiments showed that under high levels of LPS stimulation, the expression of MHC class II, CD86, and MHC II class transactivator (CIITA) in human monocytes was significantly reduced, leading to impaired antigen presentation (130). Shalova et al. treated monocytes from sepsis patients with LPS *in vitro*, and their findings indicated that the expression of genes associated with pro-inflammatory cytokines (such as *TNF-α, IL-1A, IL-1B, IL-6, IL-12A, IL-23A*) and chemokines (such as *CCL3, CCL4, CCL5, CCL20, CCL23, CXCL2, CXCL11*) were not upregulated. Similarly, gene expression related to activation-associated surface molecules (such as *CD80, CD44*) and transcription factors (such as *ATF5, NFKB1, NFKB2, REL, RELA*) were also deficient. This suggests that monocytes in sepsis patients are unable to actively respond to LPS stimulation, indicative of an immune functional defect (131). Research has also reported that hypoxia-inducible factor-1α (HIF1-α) is overexpressed in human tolerant monocytes and targets the upregulation of *PD-L1*-related gene expression, thereby mediating the suppression of T lymphocyte proliferation and activation (132). Although there are currently no universally recognized biomarkers for ET, genomic and transcriptomic analyses can partially elucidate the genetic variations associated with the onset of immune tolerance in sepsis hosts, which is helpful in distinguishing patients with immune dysfunction (133–135).

In addition to the mechanisms mentioned above, various intrinsic mechanisms contribute to secondary immunosuppression, including dysfunction in the pro-inflammatory functions of the central nervous system (136), epigenetic or transcriptional regulation (137, 138), and immune metabolic dysfunction. For instance, PBMCs exhibit reduced cytokine release capacity, and monocytes display metabolic dysfunctions such as impaired glycolysis and lipid oxidation. Additionally, mitochondrial damage within tissue and immune cells is accompanied by decreased ATP and NAD^+^ levels, reduced lactate production, and diminished oxygen consumption (139–141).

## 3 Preclinical studies of immunotherapy in the treatment of sepsis

To date, a substantial body of research has shown that immunotherapy offers some protective effects against the severity of disease, organ dysfunction, and mortality in sepsis models in animals. Although the same experimental drugs have shown significant variability in effectiveness across different sepsis modeling techniques or types of sepsis animals, some drugs effective in animal models have not yet successfully transitioned to clinical trials. Overall, the positive results from animal studies provide preliminary indications for the potential of immunotherapy in clinical trials (**Table 1**).

**Table 1:** Preclinical study of immunotherapy in the treatment of sepsis.

Immunotherapy strategies	Immune mechanism	Biological agents/drugs	Animal model	Experimental methods	Result	References
Targeted cytokines	Anti-inflammatory	Infliximab (Anti TNF-α)	CLP, septic rats	*In vivo*	Reduce serum TNF-α level, improved acute lung, liver, and kidney injury, and increased the 7-day survival rate	(142)
	Anti-inflammatory	Tocilizumab (IL-6R antagonists)	CLP, septic rats	*In vivo*	Inhibiting the activation of NF-κB, reduces the inflammatory response and oxidative stress, improved acute lung and kidney injury	(143)
	Anti-inflammatory	Anti IL-17 antibody	CLP, septic mice	*In vivo*	Increased the 7-day survival rate	(144)
	Anti-inflammatory	Anti IL-17 antibody	CLP, septic mice	*In vivo*	Inhibiting microglial cell activation and central nervous system inflammation, alleviating SAE, and improving cognitive dysfunction	(145)
	Anti-inflammatory	Anti CCR6 antibody	CLP, septic mice	*In vivo*	Inhibition γδT cell recruitment and migration, and the release of IL-17A, alleviate the infiltration of inflammatory cells in the liver	(146)
	Pro-inflammatory	rhIL-7	Peritonitis, septic mice	*In vivo*	Reduce CD4^+^T and CD8^+^T cell apoptosis and promote IFN-γ	(147)
	Pro-inflammatory	IFN- γ/Anti IL-10 antibodies	CLP, septic mice	*In vivo*	No significant effect	(148)
	Pro-inflammatory	IFN- γ	CLP, septic mice	*In vivo*	Upregulation of CD86 expression on DCs and reduction of DCs apoptosis	(149)
Targeted complement	Anti-inflammatory	Anti C5aR antibody	CLP, septic mice	*In vivo*	Weakened the accumulation of inflammatory factors, and reduced the mortality of mice	(150)
	Anti-inflammatory	Anti C5a antibody	CLP, septic mice	*In vitro*	Reduced chemotaxis of neutrophils	(151)
	Anti-inflammatory	*C5aR1* gene deficient	CLP, septic mice	*In vivo*	Increased IFN-γ while decreased IL-10	(152)
	Pro-inflammatory	Anti C1q antibody	CLP, SAE mice	*In vivo*	Protected from neuronal damage and synapse loss, and improved neurocognitive outcome	(69)
Targeted immune checkpoints	Pro-inflammatory	Anti PD-1/PD-L1/CTLA-4 antibody	CLP, primary/secondary fungal sepsis in mice	*In vivo*	Blocked lymphocyte apoptosis, increased IFN-γ, and upregulated the MHC II expression on DCs	(153)
	Pro-inflammatory	Anti PD-1 antibody	CLP, septic mice	*In vivo*	Blocked lymphocyte apoptosis, increased TNF-α and IL-6, and decreased IL-10	(154)
	Pro-inflammatory	*TIM-3* gene deficient	CLP, septic mice	*In vivo*	Reduced lymphocyte apoptosis, and restored proliferative activity, protected organ function, and reduced mortality	(155)
	Pro-inflammatory	Anti TIM-3 antibody	CLP, septic mice	*In vivo*	Reduced lymphocyte apoptosis, and relieved sepsis induced acute lung and liver injury	(156)
	Pro-inflammatory	Anti CTLA-4 antibody	CLP, septic mice	*In vivo*	Reduced lymphocyte apoptosis, and the 7-day survival rate of mice showed a significant dose-dependent effect	(157)
	Pro-inflammatory	*VISTA*-gene deficient	CLP, septic mice	*In vivo*	Reduced proportion of Tregs, compensatory upregulation of Foxp3, CTLA4, and CD25, increased inflammatory cytokines and mortality	(158)
	Anti-inflammatory	ICOS-Fc	CLP, septic mice	*In vivo*	Reduce inflammatory response, and relieved sepsis induced acute kidney and liver injury	(159)
MSCs/MSCs-Exo/MSCs-EV	Anti-apoptotic	AMSCs-Exo	CLP, se-AKI mice	*In vivo*	Activated SIRT1 signaling pathway, reduced apoptosis, inflammation, and microcirculation disorders in the kidneys	(160)
	Anti-apoptotic	HUMSCs-Exo	CLP, septic mice	*In vivo*	Targeted PINK1-PKA-NCLX axis to promote mitochondrial calcium efflux in cardiomyocytes, reduce myocardial cells apoptosis	(161)
	Anti-apoptotic	BMSCs-EV	LPS, RAW264.7	*In vitro*	Targeted BRD4/EZH2/TRAIL axis to inhibit LPS-induced inflammation and apoptosis in RAW264.7 cells	(162)
	Activate autophagy	BMSCs-Exo	CLP, se-AKI rats/ HK-2 cells	*In vivo* *In vitro*	Reduce inflammation and apoptosis by increasing autophagy in the kidneys	(163)
	Activate autophagy	AMSCs-Exo	CLP, se-AKI mice/ HK-2 cells	*In vivo* *In vitro*	Increased autophagy, mitigated kidney injury, and suppressed inflammation	(164)
	Activate autophagy	BMSCs	CLP, se-AKI rats/ HK-2 cells	*In vivo* *In vitro*	Targeted SIRT1/Parkin axis to enhanced autophagy, suppressed inflammation and apoptosis, and mitigated kidney injury	(165)
	Anti- pyroptosis	HUMSCs-Exo	LPS, BV2 cells	*In vitro*	Targeted miR-146a-5p/TRAF6 axis to increase autophagy and inhibit pyroptosis	(166)
	Anti- pyroptosis	BMSCs/BMSCs-Exo	LPS, EPCs	*In vitro*	Targeted miR-223–3p/NLRP3 axis to inhibit pyroptosis	(167)
	Anti- pyroptosis	HUMSCs-Exo	LPS, MPMs	*In vitro*	Targeted miR-378a-5p/NLRP3 axis to inhibit pyroptosis	(168)
	Anti- ferroptosis	MSCs-Exo	CCl4, ALI mice	*In vivo*	Increased SLC7A11 level and strengthening SLC7A11 stability, and inhibit ferroptosis	(169)

CLP, cecal ligation and puncture; SAE, sepsis-associated encephalopathy; rhIL-7, recombinant human IL-7; ICOS-Fc, a soluble recombinant form of ICOS; MSCs, mesenchymal stem cells; MSCs-Exo, mesenchymal stem cells-derived exosome; MSCs-EV, mesenchymal stem cells-derived extracellular vesicle; AMSCs, adipose tissue-derived mesenchymal stem cells; se-AKI, sepsis- associated acute kidney injury; HUMSCs, human umbilical cord mesenchymal stem cells; BMSCs, bone mesenchymal stem cells; EPCs, endothelial progenitor cells; MPMs, mouse peritoneal macrophages; ALI, acute liver injury.

### 3.1 Targeting cytokine

Blocking the activity of inflammatory cytokines is a fundamental strategy to inhibit acute-phase excessive inflammatory responses. For instance, *in vivo* experiments with Infliximab treatment (an anti-TNF-α antibody) have significantly reduced serum TNF-α levels in septic rats, markedly improving acute lung, liver, and kidney injuries, and increased the 7-day survival rate of rats from 0% to 37.5% (142). The IL-6 receptor antagonist Tocilizumab can inhibit NF-κB activation, significantly reducing inflammatory responses and oxidative stress levels in CLP rats, and offers protection against sepsis-induced acute lung and kidney injuries (143). Flierl and colleagues confirmed that anti-IL-17 treatment significantly increased the 7-day survival rate of CLP mice (control group: 10% vs. anti-IL-17 antibody group: 60%), and administering the treatment 12 hours later still offered some protection to the host (144). Intracerebral administration of anti-IL-17 or anti-IL-17R antibody also mitigated microglial activation and central nervous system inflammation in CLP mice by blocking the IL-17A/IL-17R pathway, alleviating sepsis-associated encephalopathy (SAE), and improving cognitive dysfunctions (145). Wan and others found that an anti-CCR6 antibody, by blocking the CCR6-CCL20 axis, inhibited the recruitment and migration of γδT cells and the release of IL-17A in CLP mice, reducing the infiltration of inflammatory cells in the liver induced by sepsis (146). In studies of immune suppression, Unsinger and colleagues tested the efficacy of recombinant human IL-7 (rhIL-7) in a peritonitis-induced sepsis model in mice. The results indicated that rhIL-7 mediated a reduction in apoptosis of CD4^+^ and CD8^+^T cells and promoted the production of IFN-γ by upregulating the expression of Bcl-2, thereby improving immune suppression in mice (147). Conversely, Murphey and others, using a combination of IFN-γ and anti-IL-10 antibody in CLP mice experiencing immune suppression, did not observe a significant improvement in bacterial clearance rates or survival rates (148). However, recent studies have shown that IFN-γ treatment in CLP mice can upregulate CD86 expression on DCs and reduce DC apoptosis, reversing the immune suppression caused by sepsis (149). These findings highlight that while supplementing or modulating cytokines—whether anti-inflammatory or pro-inflammatory—is a relatively straightforward immunomodulatory strategy, the application protocols and therapeutic outcomes require further study.

### 3.2 Targeting complements

The activated complement system plays a critical role in the transmission of inflammatory signals. For instance, studies have shown that using an anti-C5aR antibody in CLP mice significantly reduces the accumulation of inflammatory cytokines in plasma and decreases mortality (150). Another *in vitro* experiment observed that after administering an anti-C5a antibody, chemotaxis of neutrophils activated via the complement alternative pathway was significantly reduced in septic mice, helping to regulate excessive accumulation and abnormal infiltration of neutrophils in tissues (151). However, recent research indicates that C5a/C5aR also participates in anti-inflammatory signaling. Sommerfeld and colleagues observed that mice with a C5aR1 gene deficiency exhibited high levels of IFN-γ and low levels of IL-10 post-CLP (152). Beyond C5a/C5aR, other components of the complement system have also garnered attention. Chung and others discovered that hippocampal tissue expression of complement C1q was upregulated in SAE mice, mediating neuronal damage. Intracerebral injection of a specific C1q blocker significantly protected microglial cells, improving neurocognitive function impairments in mice (69).

### 3.3 Targeting immune checkpoints

Immune checkpoints (ICs) inhibit excessive activation and proliferation of immune cells, regulate inflammatory responses, prevent damage to self-tissues and organs, and promote the restoration of immune homeostasis. However, the continuous transmission of negative signaling pathways can also induce uncontrollable cell apoptosis and immune suppression. Common ICs, including PD-1/PD-L1, cytotoxic T-lymphocyte antigen-4 (CTLA-4)/CD80(CD86), B-and T-lymphocyte attenuator (BTLA)/herpes virus entry mediator (HVEM), and T-cell immunoglobulin and mucin-domain containing-3 (TIM-3)/Galectin-9 (Gal-9), are all potential therapeutic targets. Chang and colleagues reported that the individual use of anti-PD-1, anti-PD-L1, or anti-CTLA-4 antibodies could promote the release of IFN-γ by blocking lymphocyte apoptosis and upregulating the expression of MHC II on DCs, modulating the immune suppression state in mice with primary and secondary fungal sepsis (153). Similarly, Zhang and others observed that in CLP mice, the expression of PD-1/PD-L1 was upregulated on T cells, B cells, and monocytes, and that anti-PD-L1 antibodies could inhibit some lymphocyte apoptosis and exhaustion induced by sepsis, promote the release of TNF-α and IL-6, reduce the production of IL-10, and significantly improve the survival rate of the mice (154). Huang and colleagues discovered that the deletion of *TIM-3* in CD4^+^T cells in septic mice could alleviate lymphocyte apoptosis and restore their proliferative activity, thus protecting organ function and reducing mortality (155). In the same year, Liu and others observed that treatment with anti-TIM-3 in CLP mice reduced lymphocyte apoptosis and significantly alleviated sepsis-induced acute lung and liver damage (156). However, the timing and dosage of administration can lead to significant differences in treatment effectiveness. For instance, Inoue and colleagues noted an increase in CTLA-4 expression in T cells in CLP mice, and administering anti-CTLA-4 antibodies reduced sepsis-induced lymphocyte apoptosis, but the 7-day survival rate of the mice showed a clear dose dependency; higher doses of anti-CTLA-4 antibodies decreased survival rates while lower doses increased them (157). Immune checkpoint inhibitors (ICIs) play a crucial role in inhibiting persistent apoptosis and exhaustion of lymphocytes. Other novel checkpoint inhibitors such as VISTA (158) and ICOS (159) are also being developed and validated.

### 3.4 Targeting mesenchymal stem cells

Mesenchymal stem cells (MSCs) are described as balancers of the inflammatory microenvironment and immune dysregulation due to their ability to modulate the activation, maturation, proliferation, differentiation, and effector functions of various immune cells (170, 171). This modulation occurs through direct contact with target cells, the release of bioactive factors (such as cytokines, growth factors, chemokines), and paracrine pathways involving the secretion of extracellular vesicles and exosomes that contain cytokines, miRNAs, and other soluble factors. Extensive *in vivo* and *in vitro* experimental results support that MSCs and their derivatives can regulate programmed cell death (including apoptosis (160–162), autophagy (163–165), pyroptosis (166–168), and ferroptosis (169)) in immune and tissue cells, maintaining homeostasis within the host environment. They can modulate imbalanced immune responses, alleviate tissue and organ damage, improve multi-organ dysfunction, and reduce mortality. Despite satisfactory results in rodent models, the efficacy of MSCs and their derivatives in larger animal models remains unclear. For instance, Horak and colleagues observed that in pigs with peritoneal sepsis treated with MSCs, there was no significant alleviation of hemodynamic abnormalities, the systemic overactivation of inflammatory responses was unmodulated, and organ failure assessment scores continued to increase (172). Thus, further preclinical experiments are needed to determine the appropriate pathways, dosages, indications, and potential adverse reactions for the use of MSCs and their derivatives in sepsis and related diseases.

## 4 Clinical studies of immunotherapy in the treatment of sepsis

**Figure 2:**
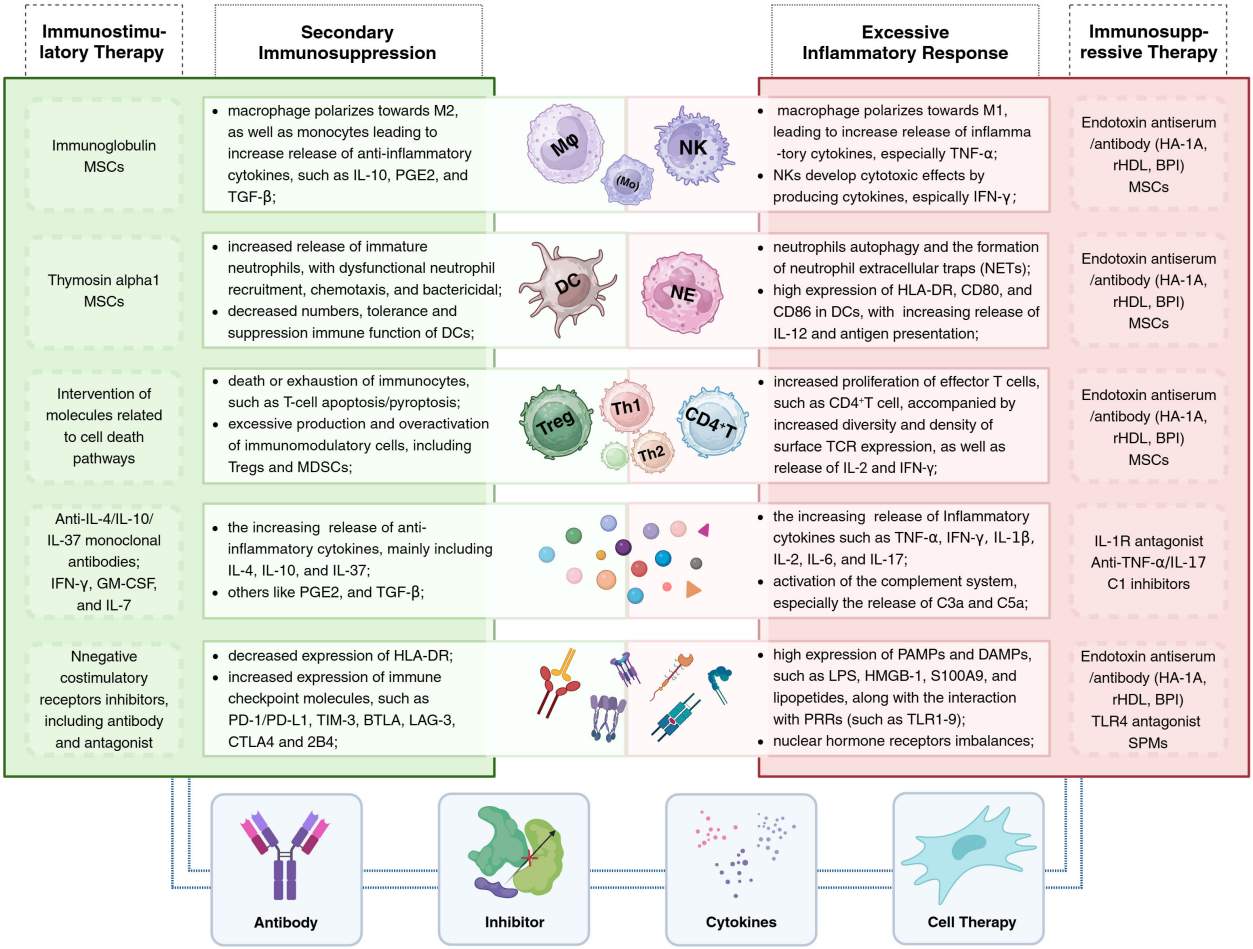
Immunotherapy for sepsis: The central portion of the illustration presents alterations in immune cells and components within the host under different immune states (with excessive inflammatory responses on the right and secondary immune suppression on the left), alongside corresponding immunotherapeutic strategies. The lower section of the illustration summarizes the four primary types of immunomodulatory agents, including antibodies, inhibitors, cytokines, and cellular therapies. BPI, bactericidal/permeability-increasing protein; HA-1A, human monoclonal anti-endotoxin antibody; rHDL, reconstituted high-density lipoprotein; SPMs, specialized pro-resolving mediators.

The clinical course of sepsis in patients is not only a race between the pathogen and the host’s immune response but also a battle between the host’s own abnormally activated inflammatory response and the subsequent anti-inflammatory response. Pathogen infection is the trigger for the onset of sepsis, while the host’s uncontrolled and disordered immune response is the key mechanism driving the progression of sepsis. Increasingly, clinical trials are attempting to modulate the components and functions of the host’s immune system to promote the restoration of immune homeostasis, yet the outcomes of these trials vary widely. To date, there are no universally recognized effective or approved immune therapies or related products for the treatment of sepsis in clinical settings. Furthermore, sepsis is a highly heterogeneous disease, which suggests that differences in immune responses among individual hosts should also be considered. Of course, as our understanding of the pathophysiological mechanisms of sepsis deepens and as biomarkers related to sepsis are continuously developed, these immune components serve not only as important indicators to assist clinicians in assessing the severity of the condition and guiding treatment but also as potential targets for immunotherapy (**Figure 2**; **Table 2**).

**Table 2:** Clinical study of immunotherapy in the treatment of sepsis and Potential biomarker.

Immunotherapy strategies	Immune mechanism	Biological agents/drugs	Disease	Population sample size	Potential biomarker	Result	References
Immuno-suppressive Therapy	Anti-inflammatory cytokines	Anakinra	sepsis	763	IL-1/IL-1R	Increased 28-day survival rate in sepsis patients with concurrent liver and gallbladder dysfunction and disseminated intravascular coagulation	(173)
		Anakinra	sepsis	280	IL-1/IL-1R	–	(174)
		rhIL-1ra	sepsis/septic shock	696	IL-1/IL-1R	No statistical difference in reducing the 28-day mortality rate	(175)
		Afelimomab	severe sepsis	2634	TNF-α, IL-6	Reduced TNF-α, IL-6, and 28-day mortality rate, attenuated the severity of organ dysfunction	(176)
		Afelimomab	sepsis	944	TNF-α, IL-6	No statistical difference in reducing the 28-day mortality rate	(177)
		Afelimomab	sepsis	48	TNF-α, IL-6	No statistical difference in reducing the 28-day mortality rate	(178)
		TNFR: Fc	sepsis	141	TNF-α	No statistical difference in reducing the 28-day mortality rate	(179)
		Allocetra™ -OTS	sepsis	10	TNF-α, IL-6	No unexpected safety findings, had immunomodulatory effects and promoted early resolution of cytokine storms	(180)
		C1-esterase inhibitor	sepsis	61	C3/C1-esterase	Reduced inflammation, and improved survival rates	(181)
		C1-esterase inhibitor	healthy volunteers	20	C4, TNF-α, IL-6	Reduced inflammation	(182)
		C1-esterase inhibitor	severe sepsis/septic shock	40	C1-esterase	Attenuated renal impairment in patients	(183)
		Ravulizumab	severe COVID-19	202	C5	No statistical difference in reducing the mortality	(184)
	Targeting PAMPs/ DAMPs related pathways	HA-1A	sepsis	543	TNF-α, IL-6	Reduced mortality in sepsis patients induced by gram-negative bacteremia	(185)
		HA-1A	sepsis	82	TNF-α, IL-6	Reduced mortality in sepsis patients induced by gram-negative bacteremia	(186)
		HA-1A	sepsis/ septic shock	2199	TNF-α, IL-6	No statistical difference in reducing the 14-day mortality	(187)
		BPI	meningococcal sepsis	393	TNF-α, IL-6	No statistical difference in reducing the mortality	(188)
		BPI	meningococcal sepsis	26	TNF-α, IL-6	No statistical difference in reducing the mortality	(189)
		Eritoran	severe sepsis	1961	TNF-α, IL-6	No statistical difference in reducing the 28-day mortality rate	(190)
Immuno-stimulatory Therapy	Supplementing immune-globulin	IgGAM	sepsis	100	–	Reduced the 28-day mortality rate	(191)
		IVIG	sepsis/severe sepsis/septic shock	2621	–	Increased survival rate (Meta analysis)	(192)
		IVIG	sepsis	6276	–	Reduced mortality, shortened hospital stay, and improved APACHE II score (Meta analysis)	(193)
	ICIs	BMS-936559	sepsis	24	_M_HLA-DR	Reduced the 28-day mortality rate	(194)
		Anti-PD-L1 antibody	sepsis	19	PD-1/PD-L1, TNF-α, IL-6	*In vitro* reduced human T cell apoptosis and IL-10, increased TNF-α and IL-6	(94)
		Nivolumab	sepsis	31	_M_HLA-DR	No unexpected safety findings or any evidence of “cytokine storm”	(195)
		Nivolumab	sepsis	13	_M_HLA-DR	No unexpected safety findings or any evidence of “cytokine storm”	(196)
		α-lactose	sepsis/ septic shock	55	Tim-3	*In vitro* reduced human NKT cell apoptosis	(197)
	Inflammatory active factors	CYT107	septic shock	27	Lymphocyte count	Increased absolute lymphocyte count and circulating CD4^+^and CD8^+^T cells by 3 to 4 times	(198)
		Tα1	severe sepsis	361	_M_HLA-DR	Reduced the 28-day mortality rate	(199)
		Tα1	sepsis	1480	–	Reduced the all-cause mortality rate (Meta analysis)	(200)
		GM-CSF	severe injury	–	_M_HLA-DR, TNF-α	*In vitro* increased _M_HLA-DR and TNF-α	(201)
		GM-CSF	severe sepsis/ septic shock	38	_M_HLA-DR	Promoted inflammation, shortened mechanical ventilation and hospitalization/ICU time	(202)
		GM-CSF	MODS patients	70	TNF-α	Increased TNF-α, reduced secondary infections in the hospital	(203)
		G-CSF/ GM-CSF	sepsis	2380	_M_HLA-DR	No statistical difference in reducing the 14-day/28-day mortality rate, and in-hospital mortality rate (Meta analysis)	(204)
		IFN-γ	sepsis	23	_M_HLA-DR	*In vivo* increased _M_HLA-DR, and *in vitro* increased TNF-α induced by LPS	(205)
		IFN-γ	healthy volunteers	18	_M_HLA-DR, TNF-α, IL-10	Increased _M_HLA-DR and TNF-α, reduced IL-10	(206)
Immuno-modulatory Therapy	MSCs	–	septic shock	9	TNF-α, IL-6	No unexpected safety findings or any evidence of “cytokine storm”	(207)
		–	severe sepsis	15	TNF-α, IL-6	No unexpected safety findings or any evidence of “cytokine storm”	(208)

Anakinra, an recombinant human interleukin-1 receptor antagonist; rhIL-1ra, recombinant human interleukin-1 receptor antagonist; Afelimomab, an anti-tumor necrosis factor F(ab’)2 monoclonal antibody fragment; TNF, tumor necrosis factor; TNFR: Fc fusion protein, a dimer of an extracellular portion of the human TNF receptor and the Fc portion of IgG1 binds; Allocetra™-OTS, early apoptotic cell; Ravulizumab, a terminal complement C5 inhibitor; HA-1A, human monoclonal anti-endotoxin antibody; BPI, bactericidal/permeability-increasing protein; Eritoran, a TLR4 antagonist; IgGAM, polyclonal IgM-enriched immunoglobulin; IVIG, intravenous immunoglobulin; ICIs, immune checkpoint inhibitors; BMS-936559, anti-PD-L1 antibody; _M_HLA-DR, monocyte human leukocyte antigen-DR; Nivolumab, an anti-PD-1 antibody; NKTs, natural killer T cells; CYT107, recombinant human IL-7; Tα1, thymosin alpha 1; G-CSF, granulocyte colony-stimulating factor; GM-CSF, granulocyte-macrophage colony-stimulating factor; MODS, multiple organ dysfunction syndrome; MSCs, mesenchymal stem cells.

### 4.1 Immunosuppressive therapy

The acute inflammatory response in sepsis patients, coupled with the encouraging results from preclinical studies of immunotherapy, has provided a preliminary basis for conducting clinical trials on immunosuppressive therapies. Although some studies indicate that administering anti-inflammatory treatments within the first few hours after the onset of sepsis can somewhat mitigate the systemic inflammatory response and protect against organ dysfunction, overall, immunosuppressive strategies have not yielded satisfactory results in clinical trials for sepsis.

#### 4.1.1 Anti-inflammatory cytokines

Numerous studies have reported that levels of pro-inflammatory cytokines such as TNF-α, IL-6, IL-18, and IFN-γ are associated with increased short-term or long-term mortality in sepsis patients, suggesting that blocking cytokine-related pathways could potentially improve host survival rates (41, 209–212). Currently, anti-IL-1 and anti-TNF-α antibodies are widely used in autoimmune diseases such as rheumatoid arthritis and ankylosing spondylitis, and their efficacy in sepsis patients has also been tested. For example, a Phase III randomized controlled trial (RCT) confirmed that Anakinra (recombinant interleukin-1 receptor antagonist/rIL-1Ra) significantly improved the 28-day survival rate of sepsis patients with concurrent hepatic and biliary dysfunction and disseminated intravascular coagulation (Anakinra group: 65.4% vs. placebo group: 35.3%). Patients with sepsis characterized by high inflammatory activity or macrophage activation syndrome (MAS) may benefit from this anti-inflammatory treatment strategy (173). Another ongoing RCT led by Kotsaki is exploring whether intravenous injection of Anakinra can improve the SOFA score and 28-day/90-day mortality rates in sepsis patients, with expected results to be published in 2025 (*ClinicalTrials.gov identifier: NCT04990232*) *(* 174). However, studies by Opal and others have shown that 72 hours of continuous intravenous infusion of rhIL-1Ra or placebo did not significantly reduce the 28-day mortality rates in patients (175). As for anti-TNF-α treatment, the results are not promising. While such therapy can reduce the concentrations of IL-6 and TNF-α in the serum of sepsis patients, its effect on reducing mortality is very limited (176–178). Furthermore, a RCT involving a dimer consisting of the extracellular portion of the human TNF receptor and the Fc portion of IgG1 (TNFR: Fc) was conducted with 141 sepsis patients randomly assigned to receive a single intravenous infusion of TNFR: Fc at doses of 0.15, 0.45, or 1.5 mg per kilogram of body weight, or a placebo. The results indicated that TNFR: Fc treatment did not reduce mortality rates, and higher doses of TNFR: Fc might be associated with an increased risk of death (179). Allocetra™-OTS (early apoptotic cells) has been demonstrated to modulate immune response. A recent Phase I clinical trial evaluated the safety and efficacy of Allocetra™-OTS in patients with sepsis. The findings indicate that this formulation is safe for patients with mild to moderate sepsis and can facilitate the early resolution of cytokine storms, thereby improving organ dysfunction and reducing ICU length of stay (180). The complement system also represents an important target for anti-inflammatory strategies. For example, C1 esterase inhibitor treatment has been shown to mitigate the systemic inflammatory response and protect renal function in sepsis patients (181–183). However, a recent Phase III clinical trial (ALXN1210-COV-305) indicated that intravenous administration of Ravulizumab (a complement C5 inhibitor) combined with supportive care did not improve clinical outcomes in hospitalized patients with severe COVID-19. Instead, there were serious IR-AEs in five patients (*ClinicalTrials.gov identifier: NCT04369469*) *(* 184).

#### 4.1.2 Targeting PAMPs/DAMPs-related pathways

Since the 20th century, numerous clinical trials have focused on neutralizing endotoxins to block the activation of inflammatory responses. However, treatments like human monoclonal anti-endotoxin antibodies (HA-1A) are applicable to infections caused by Gram-negative bacteria, and their clinical efficacy has been unstable (185–187). In addition, bactericidal/permeability-increasing protein (BPI)-related formulations have also been reported in early studies to have antimicrobial activity and neutralizing effects on endotoxins, offering some protection in severe *meningococcal* sepsis in children (188, 189). Moreover, in 2013, Steven’s team reported that Eritoran (a TLR4 antagonist) did not significantly reduce the 28-day and 12-month mortality rates in sepsis patients (*ClinicalTrials.gov identifier: NCT00334828*) *(* 190). Thus, immune therapies targeting PAMPs/DAMPs-related pathways have not yet demonstrated superiority in treatment effectiveness.

### 4.2 Immunostimulatory therapy

To date, clinical trials exploring immunosuppressive therapies for sepsis have indicated that the “theoretical” or “idealized” strategy of immunosuppression to mitigate the excessive inflammatory response in sepsis and restore host immune homeostasis is not always viable. Simultaneously, with advancing research into sepsis, a significant number of preclinical and clinical studies have observed that hosts often exhibit an excessive state of immunosuppression in the later stages of the disease. This severe immunoparalysis mediates secondary infections, subsequent deaths, or severe adverse prognoses (93, 213). In this context, there has been a shift in focus from “immunosuppressive therapy” to “immune enhancement therapy” or “immune stimulation therapy” in an effort to reverse the state of immune paralysis in sepsis hosts. The goal is to reduce apoptosis of immune cells and stimulate their proliferation and anti-inflammatory effects, enhance the release of inflammatory cells, and improve patients’ long-term survival rates.

#### 4.2.1 Immunoglobulin supplementation

Previous research has shown that sepsis patients with low levels of IgG have a significantly higher mortality rate compared to those with normal IgG levels, and that low IgG levels in sepsis patients are associated with a higher 28-day mortality (214). This suggests that intravenous immunoglobulin (IVIG) could be a valuable immune-enhancing therapy. For example, a retrospective case-control study indicated that polyclonal IgM-enriched immunoglobulin (IgGAM) reduced the 28-day mortality rates in sepsis patients compared to the control group (IgGAM treatment group: 39% vs. control group: 58%) and was an independent protective factor against 28-day mortality (OR: 0.34; 95% CI: 0.17–0.67) (191). Additionally, numerous studies have systematically reviewed the efficacy of IVIG in sepsis. For instance, Turgeon and colleagues conducted a systematic review of 20 RCTs using IVIG to treat sepsis, which suggested that IVIG treatment was closely associated with patient survival benefits compared to placebo or no intervention (risk ratio: 0.74; 95% CI: 0.62–0.89). Sepsis or septic shock patients who received a total dose of 1 gram per kilogram body weight or more (risk ratio: 0.61; 95% CI: 0.40–0.94) and those treated for more than two days (risk ratio: 0.66; 95% CI: 0.53–0.82) showed a significant correlation with improved survival rates (192). A recent meta-analysis, which included 31 RCTs, found that IVIG treatment significantly improved APACHE II scores in sepsis patients (mean difference: -1.65; 95% CI: -2.89 to -0.63), reduced hospital stay (mean difference: -4.46 days; 95% CI: -2.57 to -6.35), and decreased mortality rates (RR: 0.86; 95% CI: 0.77–0.95), particularly playing a crucial role in reducing mortality rates among adult sepsis patients (RR: 0.70; 95% CI: 0.57–0.86) (193). Although there is significant heterogeneity among the clinical trials included in the meta-analysis, including differences in population characteristics, administration regimens, types of antibody formulations, and control interventions, and varying qualities of the studies, overall, IVIG treatment has been shown to reduce the mortality rates in sepsis patients.

#### 4.2.2 Immune checkpoint inhibitors

Theoretically, in the later stages of disease marked by immune suppression, ICIs can restore T cell proliferative and effector functions by inhibiting ICs, thereby improving the host’s state of immune tolerance. For instance, a recent study found that non-surviving sepsis patients had significantly increased PD-1 expression on CD4^+^T cells, and an increased percentage of PD1^+^/CD4^+^T cells was an independent risk factor for 28-day mortality rates (OR: 1.368; 95%CI: 0.571–0.937) (215). Hotchkiss and colleagues evaluated the safety and efficacy of the PD-L1 inhibitor BMS-936559 in sepsis patients administered in single escalating doses. The results confirmed good tolerance to BMS-936559 with an overall mortality rate of 25% for all dose treatments, and significant increases in HLA-DR expression on monocytes (> 5,000 monoclonal antibodies per cell) that persisted for more than 28 days in patients receiving single doses of 300mg and 900mg (*ClinicalTrials.gov identifier: NCT02576457*) (194). Zhang and others treated lymphocytes from sepsis patients *in vitro* with anti-PD-L1 antibodies, showing that the antibody could block PD-1/PD-L1 mediated T cell apoptosis and inhibit monocyte production of IL-10 while enhancing LPS-induced levels of TNF-α and IL-6 (*ClinicalTrials.gov identifier: NCT01161745*) (94). Additionally, two studies preliminarily affirmed the safety and tolerability of Nivolumab (a PD-1 inhibitor) (*ClinicalTrials.gov identifier: NCT02960854; JAPIC identifier: JapicCTI-173600*) (195, 196). Wu and colleagues observed that upregulated expression of TIM-3 in sepsis patients mediated the apoptosis of natural killer T cells (NKTs) and was associated with disease severity and mortality, whereas *in vitro* administration of α-galactosylceramide could inhibit the apoptosis of NKTs derived from sepsis patients by blocking the TIM-3/Gal-9 pathway (197). In summary, although ICIs have shown considerable promise in preclinical studies, there is currently no direct clinical evidence to suggest that ICIs provide a definitive therapeutic effect in sepsis patients.

#### 4.2.3 Inflammatory activity factors

Direct supplementation of inflammatory cytokines or administering cytokines with immune-stimulating properties are ideal means to enhance host immunity. For instance, a study by Francois et al. showed that septic shock patients and those with severe lymphocytopenia who received 4 weeks of rhIL-7 (CYT107) treatment experienced an increase in total lymphocyte count and circulating CD4^+^and CD8^+^T cells to three to four times the baseline levels, without triggering cytokine storms, exacerbation of inflammation, or organ dysfunction (*ClinicalTrials.gov identifier: NCT02640807, NCT02797431*) (198). Thymosin alpha 1 (Tα1) is a highly conserved peptide found in the thymus, playing a key role in T cell maturation and differentiation. Its synthetic form has been approved by various regulatory agencies for the treatment of cancer and infectious diseases (216). A multicenter RCT reported that treatment with Tα1 in severe sepsis patients upregulated the expression of _M_HLA-DR, improved SOFA scores, and reduced the 28-day mortality rates from 35.0% to 26.0%, indicating that Tα1 can enhance the immune function of severe sepsis patients and reduce the 28-day all-cause mortality rates (*ClinicalTrials.gov identifier: NCT00711620*) (199). Li and colleagues conducted a systematic review of 12 clinical trials related to Tα1, indicating that Tα1 treatment could reduce the all-cause mortality in sepsis patients (pooled risk ratio: 0.68; 95%CI: 0.59–0.78). However, given the poor quality of the included studies and the small number of participants, these positive results should be interpreted with caution (200). *In vitro* administration of GM-CSF has been proven to increase the expression of _M_HLA-DR and the production of TNF-α in human monocytes stimulated with LPS (201), suggesting GM-CSF as a potential tool to enhance host immunity and reverse immune paralysis. Meisel and colleagues reported that patients with severe sepsis or septic shock who were in the immunosuppressive phase of sepsis and received GM-CSF treatment for 8 days showed a significant increase in _M_HLA-DR levels within 24 hours, returning to normal levels compared to the placebo group. Additionally, there were improvements in APACHE II scores, and a reduction in mechanical ventilation duration (*ClinicalTrials.gov identifier: NCT00252915*) (202). Another clinical trial reported that children with sepsis who developed multiple organ dysfunction syndrome (MODS) and were treated with GM-CSF showed increased production of TNF-α and a reduced risk of nosocomial secondary infections (*ClinicalTrials.gov identifier: NCT03769844*) (203). It’s indeed intriguing that Bo and colleagues, after a systematic review of 12 RCTs, found no significant correlation between GM-CSF treatment and reductions in the 14-day or 28-day mortality rates or hospital mortality rates among sepsis patients (204). This suggests that routine use of GM-CSF in sepsis patients is not supported by direct clinical evidence as a standard treatment. Research findings indicate that IFN-γ treatment can partially restore immune function in sepsis patients. Döcke and colleagues have reported that treatment with recombinant IFN-γ led to an upregulation of _M_HLA-DR expression in monocytes of sepsis patients and an increase in TNF-α secretion upon LPS stimulation *in vitro*, thereby ameliorating monocyte dysfunction (205). Similarly, Leentjens and others observed that compared to the placebo group, the IFN-γ treatment group exhibited elevated levels of TNF-α and _M_HLA-DR, and a decrease in the concentration of IL-10, suggesting an improvement in the immune response capabilities of these patients (*ClinicalTrials.gov identifier: NCT01374711*) (206).

### 4.3 Immunomodulatory therapy

Numerous preclinical studies have demonstrated that MSCs and their derivatives can protect against organ dysfunction and improve survival rates in septic animals (217). MSCs and their derivatives have unique advantages such as low immunogenicity, multi-directional differentiation potential, and the ability to modulate immune responses, providing a new option for the treatment of sepsis. However, the ethics, safety, effectiveness, and strategies for application and therapeutic mechanisms still need to be confirmed through extensive clinical trials (207, 208).

### 4.4 Registered clinical trials

As of April 19, 2024, our team entered the following keywords into *ClinicalTrials.gov*: “sepsis,” “Immunotherapy,” “Immunosuppressive therapy,” “Immunomodulatory therapy,” “Immune checkpoint,” “Checkpoint inhibitor,” “immunoregulation,” “antibody,” “Anakinra,” “Complement inhibitor,” “GM-CSF,” “Thymosin,” “MSCs,” “MSCs-Exo.” After screening, we compiled 35 registered clinical trials. These clinical trials use similar or different immunotherapy strategies but are all aimed at exploring the safety and/or effectiveness of immunotherapy in sepsis patients (**Table 3**).

**Table 3:** Clinical research on immunotherapy for sepsis.

NCT Number	Study Type	Conditions	Ages	Interventions	Outcome Measures	Status	Phase
NCT05349383	Observational	Sepsis	Child, adult, older adult	Antibody-Drug Conjugate	Sepsis-related toxicity of antibody-drug conjugate	Completed	–
NCT05996835	Interventional	SA-AKI	18 Years to 85 Years	TIN816 lyophilisate powder	AUC1–8	Recruiting	Phase 2
NCT00625209	Interventional	Septic Shock	18 Years and older	rhAPC	90-day/28-day mortality	Completed	Phase 3
NCT02960854 (195)	Interventional	Severe Sepsis	18 Years and older	Nivolumab (BMS-936558)	AEs/Immune-mediated AEs	Completed	Phase 1
NCT02025660	Interventional	Severe Sepsis	18 Years to 85 Years	poly TLR agonist (Mw)	Mortality/Hospital length of stay	Completed	Phase 2 Phase 3
NCT05267821	Interventional	Pediatric Sepsis-induced MODS	1 Day to 17 Years	Anakinra	Cumulative 28-day PELOD-2 score	Recruiting	Phase 2 Phase 3
NCT03332225	Interventional	Sepsis	18 Years and older	Anakinra	Mortality	Completed	Phase 2
NCT01766414	Interventional	Endotoxemia/ Inflammation	18 Years to 35 Years	C1-esterase inhibitor	Neutrophil phenotype and redistribution	Completed	Phase 3
NCT01275976	Interventional	Sepsis/ Inflammation	18 Years to 80 Years	C1-esterase inhibitor	Delta Interleukine-6	Terminated	Phase 3
NCT00785018	Interventional	Endotoxemia/ Inflammation	18 Years to 35 Years	C1-esterase inhibitor	Cytokines and other markers of inflammation	Completed	–
NCT04369469 (184)	Interventional	COVID-19 Severe Pneumonia	18 Years and older	Ravulizumab (C5 inhibitor)	Survival rate	Terminated	Phase 3
NCT02246595	Interventional	Severe Sepsis	18 Years and older	Monoclonal antibody CaCP29 (C5a Inhibition)	Plasma Concentration of CaCP29	Completed	Phase 2
NCT01161745 (94)	Observational	Sepsis	18 Years and older	/	/	Completed	–
NCT02576457 (194)	Interventional	Severe Sepsis/ Septic Shock	18 Years and older	BMS-936559	AEs/Immune-mediated AEs	Terminated	Phase 1
NCT00334828 (190)	Interventional	Severe Sepsis	18 Years and older	TLR4 Antagonists/eritoran tetrasodium	28-day/12-month mortality	Completed	Phase 3
NCT02640807 (198)	Interventional	Severe Sepsis	18 Years to 80 Years	IL-7	Immune reconstitution	Completed	Phase 2
NCT02797431 (198)	Interventional	Severe Sepsis	18 Years to 80 Years	IL-7	White blood count	Terminated	Phase 2
NCT00711620 (199)	Interventional	Severe Sepsis	18 Years to 85 Years	Tα1	28-day mortality, SOFA score, immune response to Tα1	Completed	–
NCT02883595	Interventional	Sepsis	18 Years and older	Tα1	Improvement of monocyte immune function, and prognosis	Completed	Phase 4
NCT02867267	Interventional	Sepsis	18 Years to 85 Years	Tα1	28-day all-cause mortality, and incidence of new onset infection within 28 days	Completed	Phase 3
NCT04901104	Observational	Sepsis	Child, adult, older adult	Tα1	1-year/3-year mortality, and recurrence rate of sepsis	Not yet recruiting	–
NCT00252915 (202)	Interventional	Sepsis	18 Years and older	GM-CSF	Reconstitution of monocytic immunity	Completed	Phase 2
NCT03769844 (203)	Interventional	Pediatric Sepsis-induced MODS	Up to 17 Years	GM-CSF	TNF-α response	Active, not recruiting	Phase 4
NCT05266001	Interventional	Pediatric Sepsis-induced MODS	1 Day to 17 Years	GM-CSF	Cumulative 28-day PELOD-2 score	Recruiting	Phase 3
NCT01374711 (206)	Interventional	Endotoxemia/ Inflammation	18 Years and 35 Years	GM-CSF/IFN-γ	The effects on immunoparalysis, and monocyte HLA-DR expression	Completed	–
NCT01653665	Interventional	Critical Illness/ Sepsis	18 Years and older	Leukine	Neutrophil phagocytosis	Completed	Phase 1 Phase 2
NCT01479114	Interventional	Neonatal Sepsis	Up to 4 Weeks	rh-GCSF	All cause mortality	Completed	Phase 4
NCT04990232 (174)	Interventional	Sepsis	18 Years and older	Anakinra, rhIFNγ	Mean total Sequential Organ Failure Assessment score, 28-day/90-day mortality	Active, not recruiting	Phase 2
NCT03633500	Interventional	Late-Onset Neonatal Sepsis	Up to 4 Months	Breastmilk	Feeding Behaviors	Completed	–
NCT03925857 (180)	Interventional	Sepsis	18 Years to 85 Years	Allocetra-OTS	AEs/Immune-mediated AEs	Completed	Phase 1
NCT03369275	Interventional	Septic Shock Sepsis	18 Years and older	Allogeneic BMSCs	The reduction in days on mechanical ventilation, or renal replacement therapy, or vasopressors	Unknown status	Phase 2
NCT05969275	Interventional	Septic Shock Sepsis	18 Years and older	Allogeneic UC-MSCs	The reduction in days on mechanical ventilation, or renal replacement therapy, or vasopressors	Recruiting	Phase 2
NCT02421484	Interventional	Septic Shock	18 Years and older	Allogeneic BMSCs	Number of adverse events as a measure of safety and tolerability	Completed	Phase 1
NCT05283317	Interventional	Septic Shock Sepsis	18 Years to 80 Years	ADMSCs	28-day mortality, length of stay in the hospital	Completed	Phase 1 Phase 2
NCT04961658	Interventional	Septic Shock	18 Years and older	GEM00220	Adverse events, maximum feasible tolerated dose	Active, not recruiting	Phase 1
NCT02899702	Interventional	Staphylococcal Infection Streptococcal Infection	1 Month to 17 Years	PRIVIGEN	Organ dysfunctions, global mortality, POPC score	Withdrawn	Phase 4

se-AKI, sepsis-associated scute kidney injury; AUC1–8, average of area under the time-corrected creatinine clearance curve from day 1 to day 8; rhAPC, recombinant human activated protein C; AEs, adverse events; MODS, multiple organ dysfunction syndrome; PELOD-2, Pediatric Logistic Organ Dysfunction score; Tα1, Thymosin alpha 1; Allocetra-OTS, early apoptotic cell.

## 5 Discussion

Despite ongoing preclinical and clinical studies further clarifying the feasibility and rationale of immunotherapy, to date, the majority of clinical trials have ended in failure, and currently, there is no universally recognized and widely applicable effective immunotherapy. We still face many challenges, including but not limited to: 1) Identifying suitable molecular targets and their corresponding biomarkers, developing engineered molecular formulations, and finding tools to monitor patient immune function; 2) Determining the safe and effective doses of related formulations, including antagonists, antibodies, cytokines, and mesenchymal stem cells; 3) Developing intervention strategies for immunotherapy, including subcutaneous, intravenous, and inhaled routes of administration; 4) The procurement and clinical management of immunomodulatory drugs to ensure their safe and effective use by the human body, as well as their appropriate handling.

Sepsis is a highly heterogeneous disease, and standardized immunotherapy only has a certain therapeutic effect on a portion of the participants, failing to benefit the majority. Therefore, in addition to classic treatment targets, some teams have used omics-based markers such as differentially expressed genes and proteins to heterogeneously categorize sepsis patients. Subsequently, they have designed and initiated stratified immunotherapies with the aim of achieving classified and precision treatment for sepsis (174, 218). For instance, Seymour et al. incorporated 29 candidate variables, including demographic characteristics, basic vital signs, markers of inflammation, and biomarkers related to organ dysfunction or injury, and employed k-means clustering to perform phenotypic analysis on sepsis patients. Their findings delineated four derived phenotypes associated with patterns of host immune response, clinical features, and treatment outcomes, enhancing our understanding of the heterogeneity in treatment effects among sepsis hosts (219). This also suggests that novel tools such as machine learning and omics analyses could be utilized for subtype or phenotype analysis in sepsis. Furthermore, considering the potential role of genetic factors in the progression of sepsis, aspects like genetic susceptibility and epigenetic modifications are garnering significant attention. For example, the genetic polymorphism rs11536889 in *TLR4* is linked to an increased risk of Gram-negative bacterial infections, as well as coagulation dysfunction, renal and hepatic dysfunction or organ failure in sepsis patients (220). Similarly, the genetic polymorphism rs11568821 in *PD-1* is associated with poor prognosis and a higher 90-day mortality rate in sepsis patients (221). Other apoptosis-related genes, such as rs2093266 in *SERPINA4*, rs1955656 in *SERPINA5* (222), and rs8094315 and rs12457893 in *BCL2* (223), have also been reported to correlate with acute kidney injury in sepsis hosts.

Indeed, the progression of sepsis is distinctly time-dependent, accompanied by unstable and unbalanced pro-inflammatory and anti-inflammatory responses and other complex immune mechanisms. These factors must be carefully considered when developing immunotherapy strategies (224). Additionally, we must not overlook the occurrence of IR-AEs, such as pulmonary infiltration and acute kidney injury (225), which can occur at any time during or after treatment and are difficult to distinguish from the recurrent infections caused by the early excessive inflammatory state or late immune suppression in sepsis.

## 6 Conclusion

Immunotherapy undoubtedly harbors significant potential for advancement in the treatment of sepsis. However, extensive research is still required to elucidate the correlations between dysfunction of immune cells related to the host in sepsis, immune suppression, chronic inflammation, and outcomes such as short-term mortality and long-term adverse prognoses. This knowledge is crucial for the development and formulation of safe, effective, and widely applicable immunotherapeutic drugs and corresponding strategies.

## Author contributions

YW: Conceptualization, Writing – review & editing, Writing – original draft. LW: Conceptualization, Writing – review & editing, Writing – original draft. HK: Writing – review & editing, Conceptualization, Supervision. YL: Project administration, Writing – review & editing. YC: Project administration, Writing – review & editing. MW: Project administration, Writing – review & editing. ZD: Supervision, Writing – review & editing.

## Glossary

**Table:**

DCs	dendritic cells
IL	Interleukin
bs-Abs	bispecific antibodies
ts-Abs	trispecific antibodies
IR-AEs	immune-related adverse events
SIRS	systemic inflammatory response syndrome
CARS	compensatory anti-inflammatory response syndrome
ET	endotoxin tolerance
CS	cytokine storm
TNF	tumor necrosis factor
IFN	interferon
PAMPs	pathogen-associated molecular patterns
DAMPs	damage-associated molecular patterns
PRRs	pathogen recognition receptors
LPS	lipopolysaccharide
MBL	mannose-binding lectin
HMGB-1	high mobility group box-1
HSP	heat shock protein
TLRs	Toll-like receptors
NLRs	nucleotide oligomerization domain-like receptors
RLRs	retinoic acid-inducible gene-I-like receptors
CLRs	C-type lectin receptors
ALRs	Absent in melanoma-2-like receptors
GDSMD	gasdermin
NETs	neutrophil extracellular traps
ROS	reactive oxygen species
RNS	reactive nitrogen species
C3	complement 3
C5a	complement 5a
PBMCs	peripheral blood mononuclear cells
MHC	major histicompatibility complex
TCR	T-cell receptor
CTLs	cytotoxic T-lymphocytes
Th	helper T cell
Tregs	regulatory T cells
CLP	cecal ligation and puncture
PD-1	programmed death receptor-1
PD-L1	programmed death ligand-1
MDSCs	myeloid-derived suppressor cells
HLA-DR	human leukocyte antigen-DR
GM-CSF	granulocyte-macrophage colony-stimulating factor
CIITA	major histocompatibility complex class II transactivator
HIF1α	hypoxia-inducible factor-1α
SAE	sepsis-associated encephalopathy
rhIL-7	recombination human IL-7
ICs	immune checkpoints
CTLA-4	cytotoxic T-lymphocyte antigen-4
BTLA	B-and T-lymphocyte attenuator
HVEM	herpes virus entry mediator
TIM-3	T-cell immunoglobulin and mucin-domain containing-3
Gal-9	Galectin-9
ICIs	immune checkpoint inhibitors
MSCs	mesenchymal stem cells
MAS	macrophage activation syndrome
HA-1A	human monoclonal anti-endotoxin antibody
BPI	bactericidal/permeability-increasing protein
IVIG	intravenous immunoglobulin
APCHE II	Acute Physiology and Chronic Health Evaluation-II score
NKTs	natural killer T cells
RCT	randomized controlled trial
MODS	multiple organ dysfunction syndrome
